# Analysis of disease comorbidity patterns in a large-scale China population

**DOI:** 10.1186/s12920-019-0629-x

**Published:** 2019-12-12

**Authors:** Mengfei Guo, Yanan Yu, Tiancai Wen, Xiaoping Zhang, Baoyan Liu, Jin Zhang, Runshun Zhang, Yanning Zhang, Xuezhong Zhou

**Affiliations:** 1 School of Computer and Information Technology and Beijing Key Lab of Traffic Data Analysis and Mining, Beijing Jiaotong University, Beijing, 100044 China; 2 Institute of Basic Research in Clinical Medicine, China Academy of Chinese Medical Sciences, Beijing, 100700 China; 3 School of Computer Science, Northwestern Polytechnical University, Xi’an, 710129 Shanxi Province China; 4 China Academy of Chinese Medicine Sciences, Beijing, 100070 China; 5 Data Center of Traditional Chinese Medicine, China Academy of Chinese Medical Sciences, Beijing, 100700 China; 6 China Academy of Chinese Medical Sciences, Guang’anmen Hospital, Beijing, 100053 China

**Keywords:** Disease comorbidity, Complex network, Network medicine

## Abstract

**Background:**

Disease comorbidity is popular and has significant indications for disease progress and management. We aim to detect the general disease comorbidity patterns in Chinese populations using a large-scale clinical data set.

**Methods:**

We extracted the diseases from a large-scale anonymized data set derived from 8,572,137 inpatients in 453 hospitals across China. We built a Disease Comorbidity Network (DCN) using correlation analysis and detected the topological patterns of disease comorbidity using both complex network and data mining methods. The comorbidity patterns were further validated by shared molecular mechanisms using disease-gene associations and pathways. To predict the disease occurrence during the whole disease progressions, we applied four machine learning methods to model the disease trajectories of patients.

**Results:**

We obtained the DCN with 5702 nodes and 258,535 edges, which shows a power law distribution of the degree and weight. It further indicated that there exists high heterogeneity of comorbidities for different diseases and we found that the DCN is a hierarchical modular network with community structures, which have both homogeneous and heterogeneous disease categories. Furthermore, adhering to the previous work from US and Europe populations, we found that the disease comorbidities have their shared underlying molecular mechanisms. Furthermore, take hypertension and psychiatric disease as instance, we used four classification methods to predicte the disease occurrence using the comorbid disease trajectories and obtained acceptable performance, in which in particular, random forest obtained an overall best performance (with F1-score 0.6689 for hypertension and 0.6802 for psychiatric disease).

**Conclusions:**

Our study indicates that disease comorbidity is significant and valuable to understand the disease incidences and their interactions in real-world populations, which will provide important insights for detection of the patterns of disease classification, diagnosis and prognosis.

## Introduction

Disease comorbidity reflects the shared molecular mechanisms or environmental factors between diseases, which would be important for improving the knowledge and management of diseases in real-world clinical settings [1–3]. It has become a major problem in treatment [4, 5], because patients with comorbidity diseases have a higher probability of hospitalization and mortality [6, 7]. Furthermore, treating patients with multiple diseases is complicate and time - consuming, as it requires consideration of longer hospital stays and more expert consultations [8, 9]. For example, when a patient suffers from multiple diseases, the treating is particularly complicate [10] because it involves uncertainty in diagnosis and treatment. If the patient takes multiple drugs at the same time, and the popular therapies with multiple drugs might cause serious side effects due to their interactions [11, 12].

Unfortunately, the patterns and the underlying mechanisms of disease comorbidity are far from fully elucidated [13]. Therefore, recently, it has become a hot research topic on disease comorbidity both from clinical observations and molecular network mechanisms. Related studies explained the mechanism of the disease comorbidities of specific diseases. For example, studies have been conducted on the comorbidities of diabetes of adults [14]. Also, some of the related studies focus on the relationship between diseases of genes, using Relative Risk and Φ-correlation to measure the correlation between two diseases [15, 16]. And there exists a study based on complex network including several diseases, for 613 nodes and 3277 edges in its network from 3,354,043 patients [17]. However, in most cases, these studies are derived from the data in Europe and United States. In addition, it is interesting that machine learning methods are useful for predicting the patterns of biomedical entities, such as genes and proteins [18–20], when utilizing the meaningful features involved in biomedical data.

Here, we utilized a large-scale clinical data and conducted our research across the full range of diseases in China population. We built a large-scale disease comorbidity network (DCN) and obtained the topological properties and their relationships by complex network measurements. In addition, we validated the shared molecular mechanisms of the clinical disease comorbidities and investigated the possibility to predict the disease occurrence using the disease trajectories by machine learning methods. The results have implications for the disease comorbidity patterns and would be helpful to manage the chronic diseases conditions in clinical settings.

## Methods

### Data sources

Our main data were derived from the hospital discharge data held in the Data Center of the China Academy of Chinese Medical Sciences, which only includes two attributes, namely diagnostic codes and the encounter sequential identifiers of patients. This made our study strictly preserved the privacy of patients.

After removing of the records with missing diagnosis codes, we obtained 8,572,137 high-quality clinical records from 453 different hospitals in China. The diagnostic codes were recorded by ICD10 (the 10th revision of the International statistical classification of diseases [21]) and we deal with them in the form of four-digit ICD10 codes for further analysis.

Disease-gene associations were derived from the MalaCards database [22], which resulted in 64,245 disease-gene associations with 3193 diseases and 8616 genes. Meanwhile, we collected the pathway information (including 325 pathways and 7253 genes) from the KEGG Database [23]. We further obtained the disease-pathway associations with 175,167 records by linking 3118 diseases and 324 pathways by combining the above two data sets.

### Data analysis methods

#### Correlation analysis

We used Relative Risk (RR) and Φ-correlation [15, 16] to measure the correlations between disease pairs. When two diseases d_i_ and d_j_ co-occur more frequently than expected by chance, we would have RR_ij_ > 1 and Φ_ij_ > 0. The RR of observing a pair of d_i_ and d_j_ affecting the same patient is given by

1 $$ {RR}_{ij}=\frac{C_{ij}N}{P_i{P}_j} $$

where C_ij_ is the number of patients affected by both diseases, N is the total number of patients in the population and P_i_ and P_j_ are the prevalence of diseases i and j. The Φ-correlation can be expressed as:

2 $$ {\phi}_{ij}=\frac{C_{ij}-{P}_i{P}_j}{\sqrt{P_i{P}_j\left(N-{P}_i\right)\left(N-{P}_j\right)}} $$

We constructed the DCN with those disease pairs with RR > 1.0 and Φ > 0.0 and the weights of disease pairs (links) were set as the co-occurrences of the corresponding diseases.

#### Network analysis

We constructed the DCN with nodes for the diseases of the comorbidity patterns extracted before. When two diseases co-occur on a patient, there’s an edge between them. The weight of the edge is the co-occurrence times which represents the relationships between the two diseases. The weights of disease pairs of which the two diseases co-occur frequently will be large.

We used four topological measurements, namely, degree, betweenness centrality (BC), clustering coefficient (CC_1_) and closeness centrality (CC_2_), to evaluate the centrality of nodes in the network. Diseases with larger degree have more relationships with other diseases in the network [23]. BC reflects the diversity of disease connection and the complexity of the disease. CC_1_ is used to measure the closeness of the neighbors to each other [24]. That is, if disease d_1_ interacts with disease d_2_ and disease d_2_ interacts with disease d_3_, the possibility of the d_1_ interacting with d_3_ is also great. CC_2_ is an index of distribution of single-source shortest distance based on node, which vividly describes the importance of node’s position in the network.

However, basic topological properties cannot fully capture the full characteristics of DCN. For example, the degree of a node only focuses on first-order connected nodes, but ignores the relationships beyond the neighboring nodes. The CC_1_ considers the closeness of adjacent nodes, but ignores the size of adjacent nodes. Therefore, we calculated the correlations between some topological measurements to identify the coupling and hierarchical patterns underlying the DCN.

### Classification methods

**Fig. 1:**
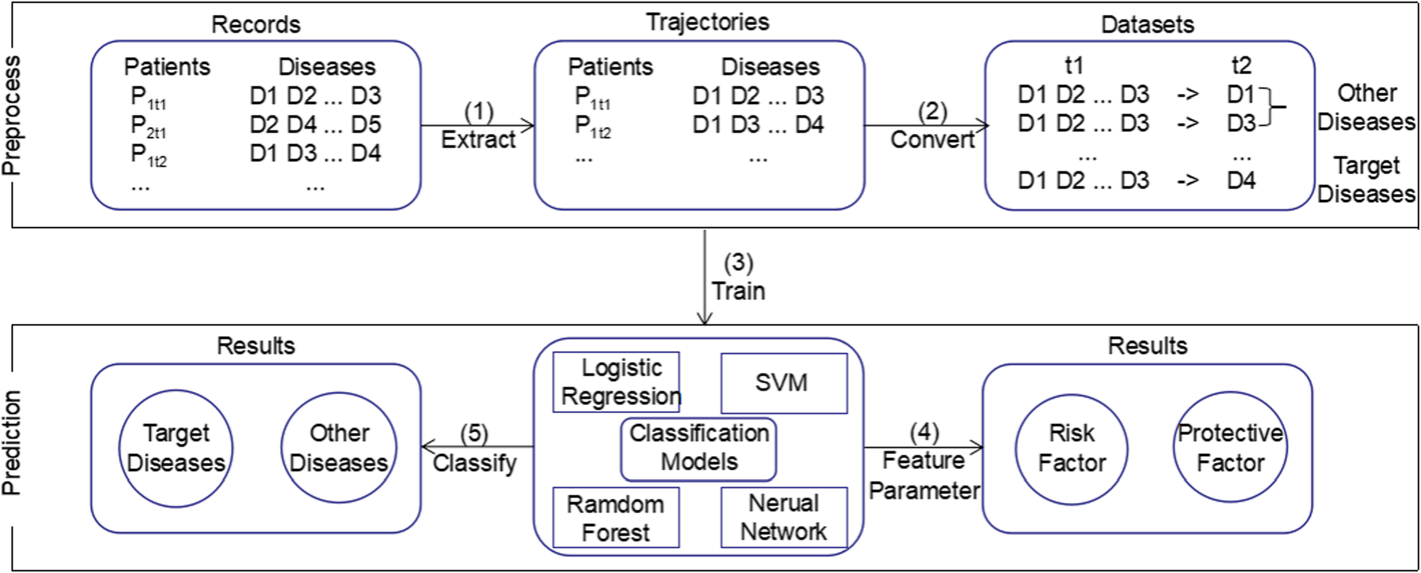
The framework to predict disease occurrence using the comorbid trajectories of patients

It is well recognized that the dynamic networks of disease comorbidities would contribute to the outcome of patients [15, 16]. Here, we investigating the feasibility of predicting disease (e.g. hypertension and psychiatric diseases) occurrence based on the comorbid trajectories of patients using four machine learning algorithms, namely Logistic Regression (LR), SVM, Random Forest (RF) and Neural Network (NN). The main framework including the preprocessing of the data set is depicted in Fig. 1.

We curated patient cases that have at least two inpatient encounters. After that, for a particular disease which is diagnosed at a specific encounter for a given patient, we would consider the past histories of diseases as the predictor variables for that particular disease. In addition, we randomly selected a set of negative samples into the benchmark for classification methods. Now we described the main steps of disease prediction task as follows.

- We extracted totally 427,939 visits from the database based on the identifiers of a patient, which includes the whole comorbid trajectories of each patient;
- Transform the data records into datasets with features and classification labels. Diseases that the patient had in the previous visits were considered as the feature (excluding the target disease), and diseases that the patient had in the current visit were considered as classification label. To predict the occurrence of a specific target disease, we set to 1 if the target disease appears, and set to 0 for the other diseases.
- Train the classification models with the preprocessed data.
- Validate the classification model (using 10-fold cross validations) and obtain the significant associated disease risk factors for a given disease.
- Use the classification model to predict the disease risks.

## Results

### Basic properties of the disease comorbidity network

**Fig. 2:**
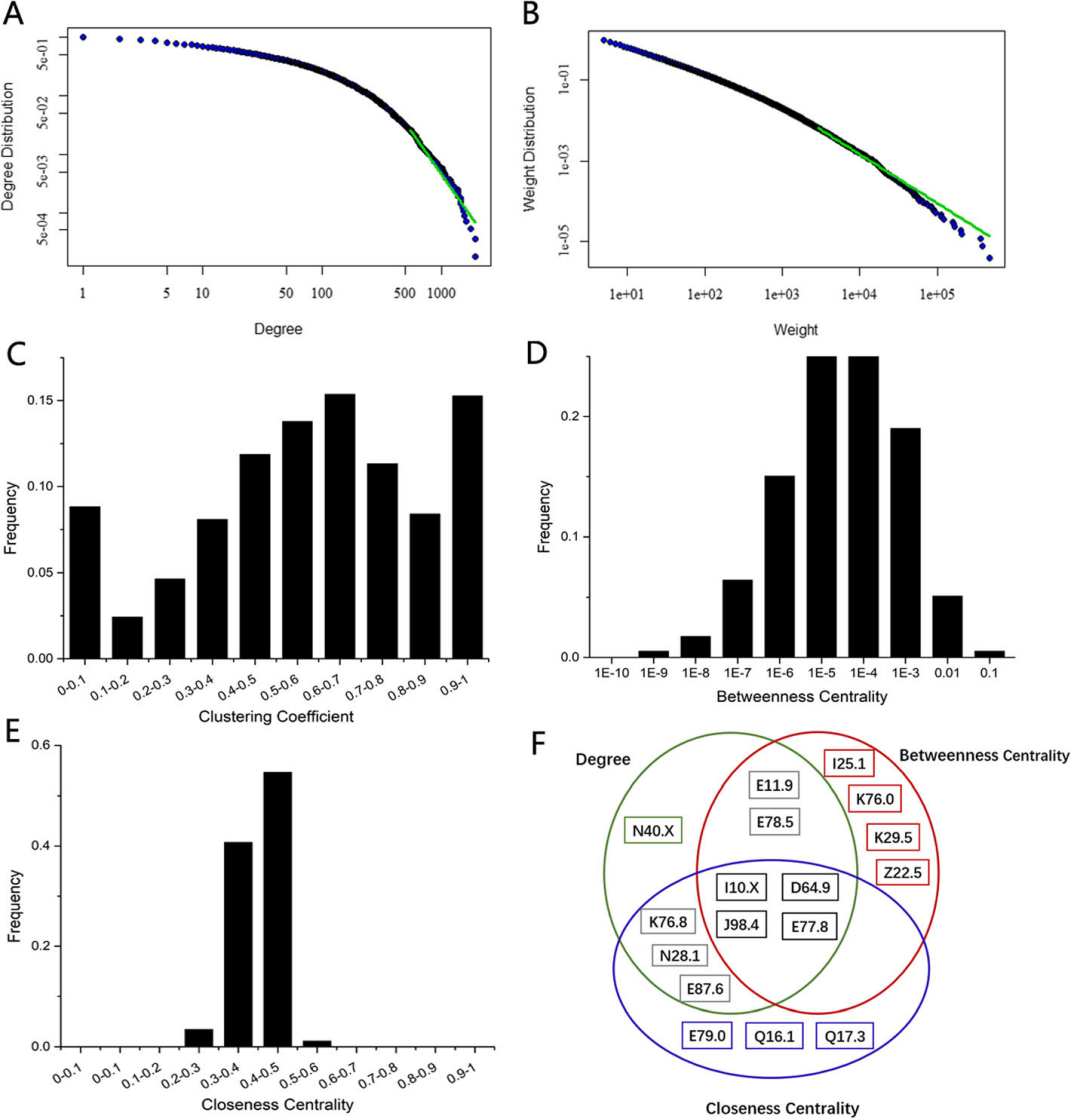
Basic properties of the network. **a** Distribution of degree. **b** Weight distribution of edges. **c** Distribution of CC_1_. **d** Distribution of BC. **e** Distribution of CC_2_. **f** The top 10 diseases with the highest degree, CC_2_ and BC, respectively

We constructed the DCN with diseases whose co-occurrence > 5, RR > 1.0 and Φ-correlation > 0.0. For these comorbid diseases filtered by the above two correlations, they actually obtained clinical meaningful relationships. For example, we found that the RR and Φ for hypertension and atherosclerotic heart disease is 2.53 and 0.2760, respectively. While the RR and Φ for hybrid asthma and atherosclerotic heart disease only got 1.3368 and 0.0002 respectively. The DCN has 5702 nodes and 258,535 edges with average degree 90.717(see Fig. 2a for degree distribution) and average edge weight 12,904.494(see Fig. 2b for weight distribution). In addition, the average path length is 2.528 and the average CC_1_ is 0.629 (see Fig. 2c for CC_1_ distribution), which indicated that DCN is a highly clustering network, with the neighbors of a disease closely connected.

The power law distribution of degree and weight (Fig. 2a and Fig. 2b) showed that DCN is a scale-free network [25], which means that some diseases (e.g. hypertension, atherosclerotic heart disease) have very high comorbidities in China population. We obtained the three disease lists, which are ranked as the top 10 diseases of degree, betweenness centrality and CC_1_ (Fig. 2f). It showed that hypertension, anaemia, other disorders of lung and other disorders of glycoprotein metabolism are the top 4 diseases included in all these rank lists.

### Hierarchical modular structures of disease comorbidity network

**Fig. 3:**
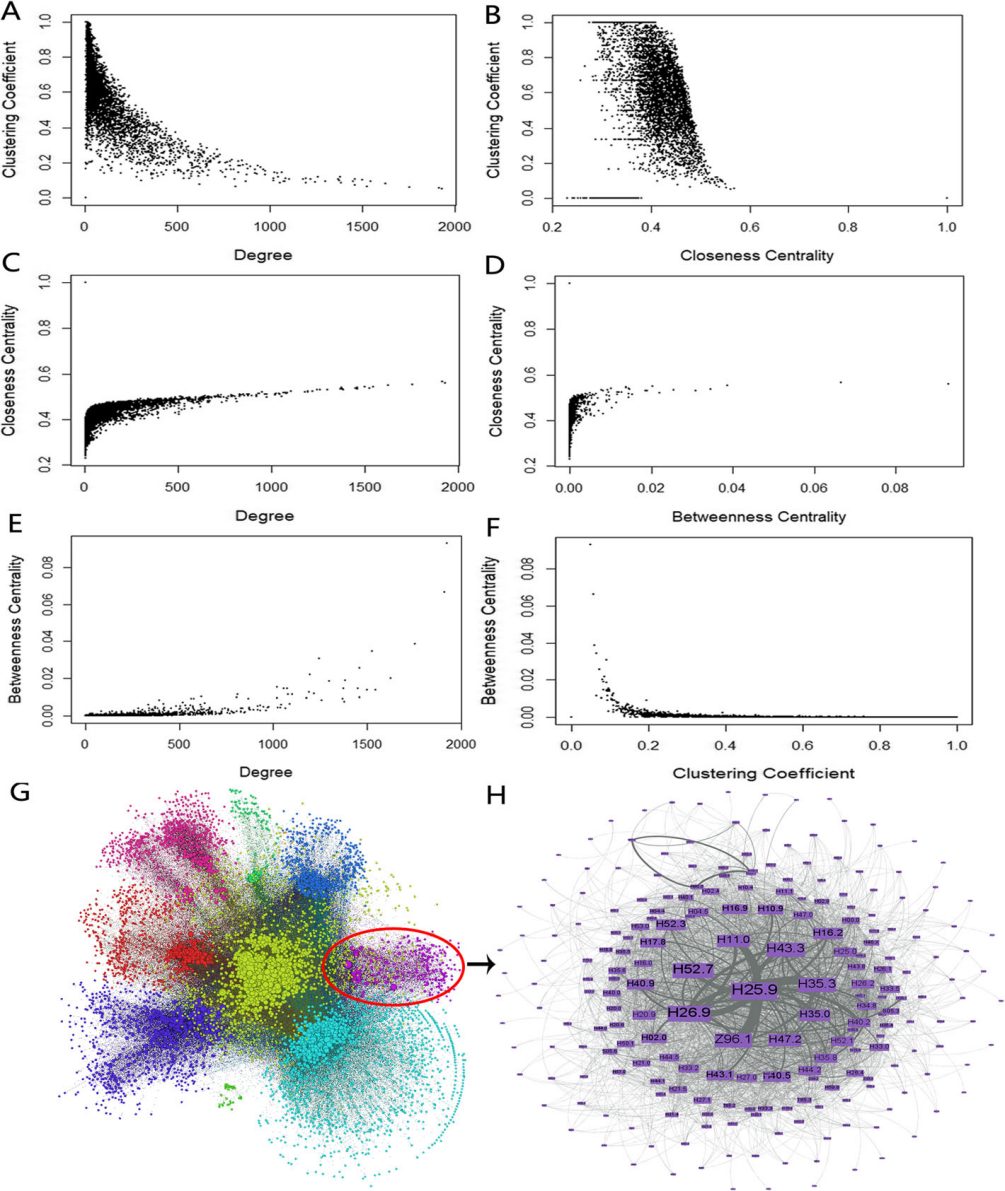
The relationship between topological properties and the network structure. **a** Degree and CC_1_; **b** CC_2_ and CC_1_; **c** Degree and CC_2_; **d** BC and CC_2_; **e** Degree and BC; **f** CC_1_ and BC; **g** Modules in the network; **h** One specific disease comorbidity module in the network

To identify the more elucidated patterns in the DCN, we calculated the correlations between several pairs of network topological measurements (Fig. 3a-f). We found that there exists negative correlation between degree and CC_1_ (Pearson correlation coefficient (PCC) = − 0.398, see Fig. 3a) in DCN, which indicated that DCN is a hierarchical modular network [26]. Furthermore, consistently, we found that there exists negative correlation between CC_1_ and CC_2_ (PCC = -0.155, see Fig. 3b). These two results showed that in DCN, the neighbors of diseases located in the center of the network (easier to get to other nodes) have large diversity and diseases with less CC_2_ tend to occur simultaneously with diseases in the same module.

Furthermore, the positive correlation between CC_2_ and degree (PCC = 0.596, see Fig. 3c) indicates that the data is reliable, because both the degree and close centrality reflect the centrality of a node.

The BC can reflect the diversity of disease connotation. There exists negative correlation between BC and CC_1_ (PCC = -0.181, see Fig. 3f), which shows that neighbors of the disease with large CC_1_ are not connected closely as a hub node. For example, as a hub node in DCN, hypertension has high BC and degree (BC = 0.093, degree = 1926), which reflects its diverse mechanisms and comorbid phenotypes. Also, the relationships between its neighbors are sparse (CC_1_ = 0.051), which indicate that there exist potential subtypes of hypertension disorder. For disorders of choroid (H31.8), its BC is 0. It has much fewer neighbors (degree = 12) but is more closely related to them than hypertension (CC_1_ = 1). That is to say, the number of the comorbidity diseases of the disease is few, but their relationship between their comorbid diseases is strong.

### Disease comorbidity communities

To identify the disease comorbidity groups from the DCN, we applied BGLL community detection method [27] to find the communities, which resulted in 10 communities with denser comorbidity links between the diseases other than random expectations (see Fig. 3g-h). There are both homogeneous and heterogeneous comorbidity diseases in the same communities. Meanwhile, there exist branching relationships between categories. For example, a specific disease comorbidity community (see Fig. 3h), includes 157(accounting for 74.8%) eye related diseases, which are caused by cataracts (H25-H26) and also contains 53(25.2%) diseases from other categories. Ocular comorbidity diseases are common in people with cataracts in real-world clinical settings [28]. This would be insightful for the refinement of disease classification.

We found several common disease comorbidity patterns from 5702 diseases, such as diabetes and obesity [29]. Hypertension occurs most frequently in the DCN. It has significant disease comorbidity patterns with arteriosclerosis heart disease (RR = 2.53, co-occurrence = 475,649), diabetes (RR = 2.56, co-occurrence = 383,436), cerebral infarction (RR = 2.70, co-occurrence = 367,144), hyperlipidemia (RR = 2.24, co-occurrence = 205,967) and heart failure (RR = 5.97, co-occurrence = 201,495). This is consistent with the popular prevalence of hypertension, which can lead to a variety of complications (e.g. cardiovascular disease [30, 31], diabetes [32, 33], renal failure [34] and obesity [35, 36]) and cause damage to organs, such as the heart, brain and kidneys. It is well known that hypertension is a serious threat to the human health. The treatment of hypertension can reduce the occurrence of cardiovascular disease and alleviate its symptom. We also find other disease comorbidity patterns, such as Alzheimer disease and atherosclerotic heart disease, which can be supported by the evidence that cardiovascular and arterial disease is considered an important risk factor for Alzheimer’s disease [37]. It is similar for the findings of the relationship of diabetes and senile cataracts. Discovering these disease relationships is beneficial to the prevention of concurrent disease while discovering the primary disease.

### Shared molecular mechanisms of disease comorbidities

To validate the correlation between disease comorbidity and their underlying shared molecular mechanisms [16] in our data, we calculated PCC between the number of shared genes and pathways and the strength of disease comorbidity (RR and Φ-correlation) in 258,543 disease pairs. We found that although the correlation is weak, there does exist significant positive correlation between comorbid diseases and their underlying molecular mechanisms (Table 1), which indicates that if two diseases share genes or pathways, it will tend to have disease comorbidities.

**Table 1:** PCC between the disease comorbidity and shared molecular mechanisms

	Shared genes	Shared pathways
RR	0.05312(*P* < 2.2e-16)	0.008511 (*P* = 0.01193)
Φ-correlation	0.23688(*P* < 2.2e-16)	0.037891 (*P* < 2.2e-16)

**Fig. 4:**
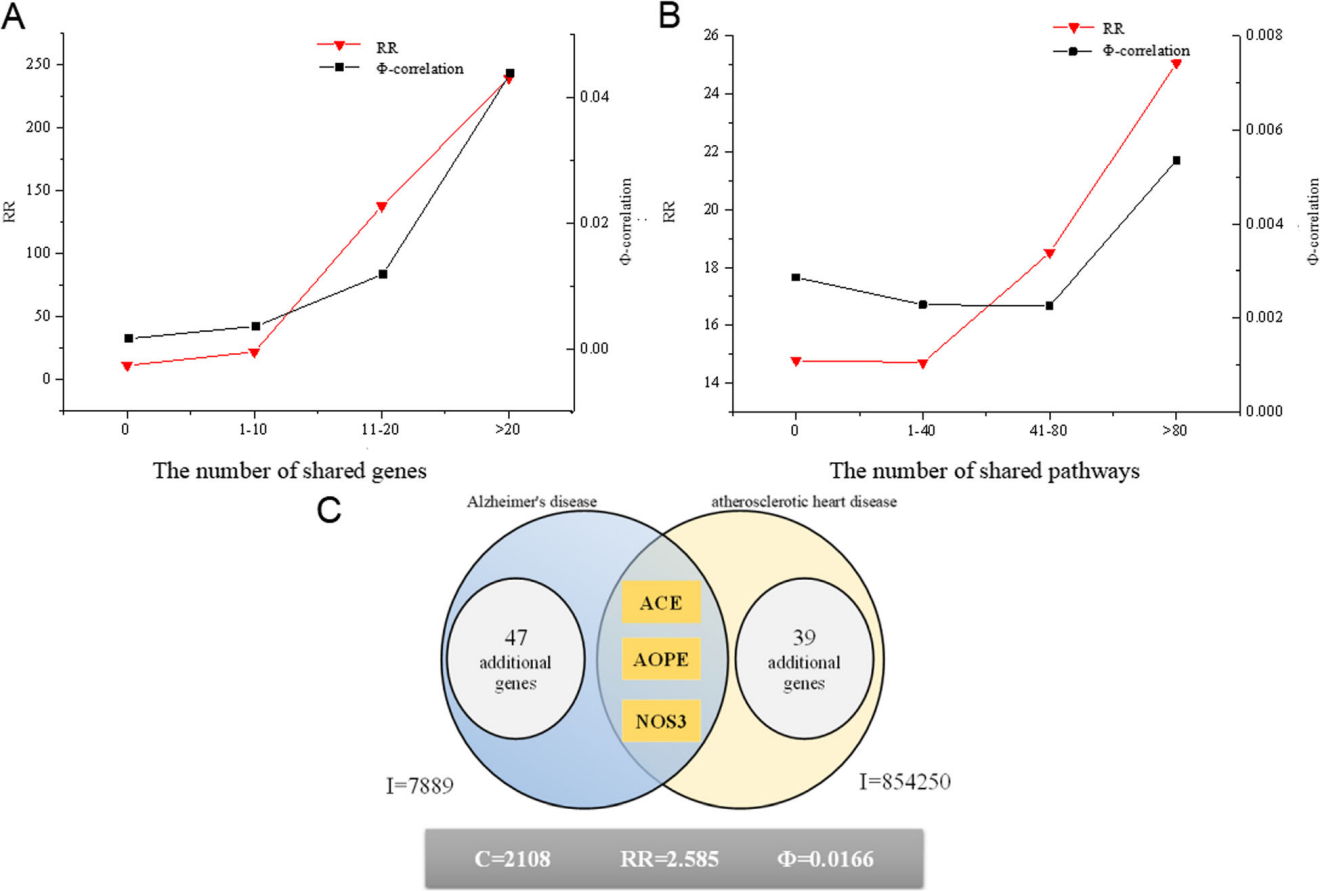
The shared molecular mechanisms of disease comorbidity. **a** The relationship between shared genes and intensity of disease comorbidity **b**. The relationship between shared pathways and intensity of disease comorbidity **c**. Disease comorbidity of Alzheimer’s Disease and Arteriosclerotic Heart Disease

In addition, we observed that the degree of disease comorbidity would be higher as their molecular correlation (shared genes and pathways) increased (see Fig. 4a and b). With the increase of molecular correlation, the degree of disease comorbidity gradually increases. Compared with the two diseases that do not share genes, the degree of diseases comorbidity of diseases sharing more than 20 genes has increased nearly five times. That is to say, the more genes the two diseases shared, the more likely there exists a disease comorbidity relationship. As the number of shared pathways increases, the comorbidity relationship becomes stronger. However, the impact is relatively weak, and there is a downward trend in the first two intervals. Therefore, we need to prevent the disease from happening while treating its comorbidity disease if they have shared genes or pathways.

We further applied two commonly used similarity measures, namely Jaccard and Cosine measures, to identify the relationship between shared genes and pathways. We calculated the similarity and PCC between them*.* The positive correlation of them (see Table 2) indicates that if the similarity of two diseases increases, the number of shared genes and pathways will increase as well.

**Table 2:** PCC between disease similarity and molecular mechanisms

	Jaccard	Cosine
Shared genes	0.1166 (*P* < 2.2e-16)	0.1312 (*P* < 2.2e-16)
Shared pathways	0.0705 (*P* < 2.2e-16)	0.0826 (*P* < 2.2e-16)

Furthermore, we found that several pairs of diseases not only have correlation at the gene level, but also show important disease comorbidity relationship, such as Alzheimer’s disease and atherosclerotic heart disease (see Fig. 4c). There is a significant disease comorbidity relationship between them (RR = 2.585, Φ-correlation = 0.017), and they have shared genes (ACE, AOPE and NOS3). This shows that the existence of shared genes may lead to the co-occurrence of two diseases, which may be the direct reason of the disease comorbidity of them.

### Disease prediction using the comorbid trajectories of patients

To investigate the possibility of using disease comorbid trajectories to predict disease occurrence, we extracted 27,000 cases from our database and generated two benchmark data sets for two disease cases, namely hypertension and psychiatric diseases to demonstrate the feasibility (see Table 3). It is noted that the coupled negative records were randomly selected from our database. We applied 4 machine learning methods (see Table 4 for detailed parameters) to predict the disease occurrence according to the previous diseases of a given patient.

**Table 3:** Positive and negative sample distribution in the data set

Data set	Positive	Negative	Total
Hypertension	10,000	10,000	20,000
Psychiatric diseases	3500	3500	7000

**Table 4:** Settings and parameters for classification methods

Methods	Setting
LR	using L2 regularization norm regularization intensity = 1
SVM	using the linear kernel function penalty parameter of the error term = 10
RF	Decision tree = 180 Bootstrap Sample oob_score = true Feature = Gini coefficient
NN	Using multilayer feedforward neural network learning rate = 0.001 maximum number of iterations = 200 two hidden layers randomly optimizing the size of mini batches

Finally, we found that the prediction results of the 4 classification models on two disease datasets (see Table 5) are acceptable. Among the two data sets, LR had the highest accuracy (0.6193 for hypertension and 0.6478 for psychiatric diseases) and NN had the lowest accuracy (0.5919 for hypertension and 0.6306 for psychiatric diseases), and RF has the highest recall (0.7534 for hypertension and 0.7358 for psychiatric diseases). Altogether, RF has the best F1-score in those four methods (0.6689 for hypertension and 0.6802 for psychiatric diseases). RF reaches the best result because it classified samples in a more interpretative way than NN and more complicated than LR. Also, with the limitation of simple networks and poor interpretability, NN may not be suitable for this task.

**Table 5:** The classification results of the four models on hypertension and psychiatric diseases

Model	Hypertension			Psychiatric diseases		
	Precision	Recall	F1-score	Precision	Recall	F1-score
LR	**0.6193 ±** 0.0140	0.6837 **±** 0.0183	0.6498 **±** 0.0127	**0.6478 ±** 0.0197	0.6900 **±** 0.0194	0.6681 **±** 0.0178
SVM	0.6038 **±** 0.0168	0.7199 **±** 0.0152	0.6567 **±** 0.0138	0.6334 **±** 0.0207	0.7041 **±** 0.0192	0.6668 **±** 0.0179
RF	0.6034 **±** 0.0239	**0.7534 ±** 0.0373	**0.6689 ±** 0.0059	0.6386 **±** 0.0293	**0.7358 ±** 0.0774	**0.6802 ±** 0.0297
NN	0.5919 **±** 0.0151	0.6166 **±** 0.0118	0.6038 **±** 0.0084	0.6306 **±** 0.0225	0.6534 **±** 0.0275	0.6415 **±** 0.0219

The highest values of the related measures are showed in bold values

In addition, we found the risk diseases that lead to hypertension and psychiatric diseases according to the coefficient in LR, SVM and RF (see Table 6). For example, in the RF method, hypertensive heart disease with (congestive) heart failure (I11.0) is one of the risk factors of hypertension. If it appeared on a patient, it will be possible that hypertension appears. Previous study held the view that hypertension is the common reason of heart failure, and 50% patients with hypertension may have heart failure as comorbidities [38]. Also, hypertension may cause effect to eyes and lead to a series of eye diseases (such as H35.0 and H52.3) [39]. Similarly, as one of the risk factors of psychiatric diseases, palpitations (R00.2) appear frequently under the influence of the side effect of anti-psychotic drugs and effects of patients’ own heart and disease [40]. For SVM, Aortic (valve) stenosis with insufficiency (I35.2) is the risk factor. It appears with hypertension frequently and several studies counted the comorbidity pattern of them (morbidity = 20%~ 68% [41, 42]). Pulmonary embolism with mention of acute cor pulmonale(I26.0), other specified inflammatory liver diseases(K75.8) and alcoholic liver disease, unspecified(K70.9) are risk factors. Due to the influence of anti-psychotic drugs, the burden on the liver will increase and the liver function will deteriorate. However, without the use of psychotropic drugs, the mood of patients will also cause liver failure. Therefore, patients with psychiatric diseases are more likely to suffer from lung disease, liver disease and heart disease complications than ordinary patients [43]. Similarly, Atherosclerotic heart disease (I25.1) as the common cardiovascular diseases [31, 32] have the disease comorbidity relationships, which is similar to diabetes [33, 34]. In summary, although some evident cofounders, such as the missing recording of target diseases in the clinical settings, would involve target disease induced comorbidities conversely as the risk diseases, we obtained acceptable prediction results for the two demonstrating diseases. In addition, we found that several common diseases, such as, heart failure, cerebral infarction and lung disease, were filtered by the three classification methods as the main risk factors for the targeting disorders (see Table 6). However, high rates of predicted risk diseases were different among the three methods, which is partially due to the mutual dependences between the risk diseases. For example, although the two risk diseases: E53.9(Vitamin B deficiency) and H35.0(a type of retinopathy and retinal disorders) predicted by SVM and LR respectively are different, they are two well recognized disorders with physio-pathological associations. Meanwhile, these predicted different features also means that it could be combined by more systematic frameworks to obtain more improved results in the future work.

**Table 6:** Important diseases for hypertension and psychiatric diseases in classification method0073

	LR			SVM			RF		
	ICD10	Disease	Regression coefficient	ICD10	Disease	Feature weights	ICD10	Disease	Importance
Hypertension	H35.0	Background retinopathy and retinal vascular changes	1.5174	I35.2	Aortic (valve) stenosis with insufficiency	1.6055	Z51.1	Chemotherapy session for neoplasm	0.0326
	A15.6	Tuberculous pleurisy, confirmed bacteriologically and histologically	1.4360	A15.6	Tuberculous pleurisy, confirmed bacteriologically and histologically	1.5705	I25.1	Atherosclerotic heart disease	0.0274
	I11.0	Hypertensive heart disease with (congestive) heart failure	1.3145	E53.9	Vitamin B deficiency, unspecified	1.4400	B18.1	Chronic viral hepatitis B without delta-agent	0.0188
	H52.3	Anisometropia and aniseikonia	1.2809	E15.X	Nondiabetic hypoglycaemic coma	1.4358	I63.9	Cerebral infarction, unspecified	0.0184
	R10.1	Pain localized to upper abdomen	1.2530	M89.9	Disorder of bone, unspecified	1.3565	I50.9	Heart failure, unspecified	0.0184
Psychiatric diseases	R00.2	Palpitations	1.6927	I63.1	Polydipsia	1.5527	Z51.1	Chemotherapy session for neoplasm	0.0323
	R62.8	Other lack of expected normal physiological development	1.4442	I26.0	Pulmonary embolism with mention of acute cor pulmonale	1.5192	C34.9	Bronchus or lung, unspecified	0.0250
	R79.8	Other specified abnormal findings of blood chemistry	1.3983	K75.8	Other specified inflammatory liver diseases	1.5137	I63.9	Cerebral infarction, unspecified	0.0236
	I63.1	Cerebral infarction due to embolism of precerebral arteries	1.3883	K70.9	Alcoholic liver disease, unspecified	1.4400	G30.9	Alzheimer disease, unspecified	0.0210
	E11.0	Type 2 diabetes mellitus	1.3871	R79.8	Other specified abnormal findings of blood chemistry	1.3510	C78.7	Secondary malignant neoplasm of liver and intrahepatic bile duct	0.0189

## Discussion

Disease comorbidity holds significant medical insights and has its underlying molecular mechanisms [15, 16], which has been a hot research topic in both clinical and network medicine fields [17]. However, most results were mainly derived from the analysis of the clinical data in Europe and United States. Due to the influence from environment factors, ethnicity and social factors to disease patterns, it is important to investigate the disease comorbidity patterns in large-scale populations in China [14, 44].

Our research is carried out across 5702 diseases in 22 categories and 8,572,137 patients with full range of the age groups. Therefore, the range of our study is more extensive in both data and scale than most previous studies in China population, which has great significance for the study of disease comorbidities. We focus on the DCN and analyzed the correlation of diseases in the network. Furthermore, we have investigated the relationships between the topological characteristics of DCN network and found biomedical meaningful patterns (i.e. the hierarchical structures of DCN). In terms of disease prediction, the prediction results are greatly influenced by the data, so the differences among countries, regions and populations in the data will also become obvious. It is significant for us to use China’s disease comorbidity data to predict disease occurrence and detect the risk factors from comorbid disease conditions.

The major limitation of our research is that the recording of diseases in clinical data would prone to incomplete diagnoses. Because clinical practitioners would tend to record the diseases that they primarily treated rather than all the diseases of patients. This would particularly induce cofounders to our prediction results and make them vulnerable. Many factors (such as age, physical condition and treatment methods, etc.) will affect the occurrence and development of a disease, which have not been incorporated in our data set. Moreover, our prediction experiments are limited to the classical supervised learning methods, which mostly provides a feasible demonstration of the prediction of disease occurrence with comorbid trajectories. In the future, we will carry out more dedicated machine learning models with more systematic clinical features, such as deep learning, to obtain more powerful predictors, which might result in practical prediction applications using disease comorbidities.

## Conclusion

We constructed a disease comorbidity network derived from millions of electronic medical records with diagnostic codes in China and found interesting topological patterns (e.g. high clustering and hierarchical modularity) for this network. Furthermore, we identified clinical meaningful disease comorbidity communities and revalidated the shared underlying molecular assumptions of disease comorbidity. Finally, by formulating the disease comorbid trajectories into a binary classification problem, we investigated the feasibility of predicting the disease occurrence using only the temporal relationships between disease phenotypes.

## Data Availability

Not applicable.

## Abbreviations

BC: Betweenness centrality
CC_1_: Clustering coefficient
CC_2_: Closeness centrality
DCN: Disease comorbidity network
ICD10: The 10th revision of the International statistical classification of diseases
LR: Logistic regression
NN: Neural network
PCC: Pearson correlation coefficient
RF: Random forest
RR: Relative risk

## References

[1] Capobianco E, Lio P (2013). Comorbidity: a multidimensional approach. Trends Mol Med.

[2] Radner H, Yoshida K, Smolen JS (2014). multimorbidity and rheumatic conditions-enhancing the concept of comorbidity. Nature reviews. Rheumatology.

[3] Rubioperez C, Guney E, Aguilar D (2017). Genetic and functional characterization of disease associations explains comorbidity. Sci Rep.

[4] Hu JX, Thomas CE, Brunak S (2016). Network biology concepts in complex disease comorbidities. Nat Rev Genet.

[5] Bragina EY, Freidin MB, Babuskina NP (2016). The analysis of associations between cytokine network genes and inverse co-morbidity of ronchial asthma and tuberculosis. Biomed Genet Genom.

[6] Steven M, Haffner, Lehto S, Tapani R (1998). Mortality from coronary heart disease in subjects with type 2 diabetes and in nondiabetic subjects with and without prior myocardial infarction. N Engl J Med.

[7] Weiner DE, Tighiouart H, Stark PC (2004). Sarnak, kidney disease as a risk factor for recurrent cardiovascular disease and mortality. Am J Kidney Dis.

[8] Starfield B, Lemke KW, Bernhardt T (2003). Comorbidity: implications for the importance of primary care in ‘case’ management. Ann Fam Med.

[9] Struijs JN, Baan CA, Schellevis FG (2006). Comorbidity in patients with diabetes mellitus:impact on medical health care utilization. BMC Health Serv Res.

[10] Gijsen R, Hoeymans N, Schellevis FG (2001). Causes and consequences of comorbidity: a review. J Clin Epidemiol.

[11] Levin A, Djurdjev O, Barrett B, Thompson C (2001). Cardiovascular disease in patients with chronic kidney disease: getting to the heart of the matter. Am J Kidney Dis.

[12] Von Lueder TG, Atar D (2014). Comorbidities and polypharmacy. Heart Fail Clin.

[13] He F, Zhu G, Wang YY (2016). PCID: a novel approach for predicting disease comorbidity by integrating multi-scale data. IEEE/ACM Transact Comput Biol Bioinf.

[14] Chen H, Zhang Y, Wu D (2016). Comorbidity in adult patients hospitalized with type 2 diabetes in Northeast China: an analysis of hospital discharge data from 2002 to 2013. Biomed Res Int.

[15] Hidalgo CA, Blumm N, Barabási A (2009). A dynamic network approach for the study of human phenotypes. PLoS Comput Biol.

[16] Park Juyong, Lee Deok‐Sun, Christakis Nicholas A, Barabási Albert‐László (2009). The impact of cellular networks on disease comorbidity. Molecular Systems Biology.

[17] Chen Y, Xu R (2014). Network Analysis of Human Disease Comorbidity Patterns Based on Large-scale Data Mining. International Symposium on Bioinformatics Research and Applications.

[18] Shen Z, Bao W-Z (2018). Recurrent neural network for predicting transcription factor binding sites. Sci Rep.

[19] Yi H-C, You Z-H (2018). A deep learning framework for robust and accurate prediction of ncRNA-protein interactions using evolutionary information. Mol Ther Nucleic Acids.

[20] Deng S-P, Lin Z (2016). Predicting hub genes associated with cervical cancer through gene co-expression networks. IEEE/ACM Trans Comput Biol Bioinform.

[21] Organization, W H (1992). ICD-10: International Statistical Classification of Diseases and Related Health Problems 10th Rev. World Health Org.

[22] Rappaport N, Nativ N, Stelzer G (2013). MalaCards: an integrated compendium for diseases and their annotation. Database (Oxford).

[23] Kanehisa M, Goto S (1999). KEGG: Kyoto encyclopedia of genes and genomes. Nucleic Acids Res.

[24] Han J, Pei J, Yin Y (2000). Mining frequent patterns without candidate generation. ACM SIGMOD Rec.

[25] Newman MEJ (2003). The structure and function of complex networks. SIAM Rev.

[26] Ravasz E, Barabási AL (2003). Hierarchical organization in complex networks. Phys Rev E.

[27] Chaturvedi P, Dhara M, Arora D (2012). Community detection in complex network via BGLL algorithm. Int J Comp Appl.

[28] Pham TQ, Wang JJ, Rochtchina E (2004). Systemic and ocular comorbidity of cataract surgical patients in a western Sydney public hospital. Clin Exp Ophthalmol.

[29] Liu Y, Congdon NG, Fan H (2007). Ocular comorbidities among cataract-operated patients in rural China: the caring is hip Study of Cataract Outcomes and Uptake of Services (SCOUTS). Ophthalmology.

[30] Evans JM, Newton RW, Ruta DA (2000). Socio-economic status, obesity and prevalence of Type 1 and Type 2 diabetes mellitus. Diabet Med.

[31] Dzudie A, Kengne AP, Mbahe S (2008). Chronic heart failure, selected risk factors and co-morbidities among adults treated for hypertension in a cardiac referral hospital in Cameroon. Eur J Heart Fail.

[32] Conti CR (2001). Diabetes, hypertension, and cardiovascular disease. Clin Cardiol.

[33] Channanath AM, Farran B, Behbehani K (2013). State of Diabetes,Hypertension, and Comorbidity in Kuwait: Showcasing the Trends as Seen in Native Versus Expatriate Populations. Diabetes Care.

[34] Tripathy JP, Thakur JS, Jeet G (2017). Prevalence and determinants of comorbid diabetes and hypertension: Evidence from non communicable disease risk factor STEPS survey, India. Diabetes Metab Syndr.

[35] Sarafidis PA, Li S, Chen SC (2008). Hypertension awareness, treatment, and control in chronic kidney disease. Am J Med.

[36] Lukas A, Kumbein F, Temml C (2003). Body mass index is the main risk factor for arterial hypertension in young subjects without major comorbidity. Eur J Clin Investig.

[37] Uretsky S, Messerli FH, Bangalore S (2007). Obesity paradox in patients with hypertension and coronary artery disease. Am J Med.

[38] Sun G, Huang G (2016). Treatment strategy of hypertension with heart failure. Adv Cardiovasc Dis.

[39] Gao Y, Wei Q (2008). Hypertensive ophthalmopathy. Int J Ophthalmol.

[40] Yi W, Wei W, Liu Y (2014). Discussion on the experience of applying traditional Chinese medicine to psychiatric patients with palpitation syndrome. Medical Frontier.

[41] De Simone G (2010). The difficult clinical management of the combination of hypertension with aortic stenosis. J Hypertens.

[42] Cao X, Ma J (2016). Influence of hypertension on diagnosis and treatment of aortic stenosis and countermeasures. J Cardiovasc Surg.

[43] Sokal J, Messias E, Dickerson FB (2004). Comorbidity of medical illnesses among adults with serious mental illness who are receiving community psychiatric services. J Nerv Ment Dis.

[44] Liu J, Ma J, Wang J (2016). Comorbidity analysis according to sex and age in hypertension patients in China. Int J Med Sci.

